# A Monocentric Retrospective Study about the Correlation between Histology and Cytology of Thyroid Indeterminate Nodules Classified as TIR 3A and TIR 3B, according to 2014 Italian Consensus for Classification and Reporting of Thyroid Cytology

**DOI:** 10.1155/2019/3932721

**Published:** 2019-10-08

**Authors:** Francesco Quaglino, Giulia Arnulfo, Sergio Sandrucci, Claudio Rossi, Valentina Marchese, Roberto Saracco, Stefano Guzzetti, Stefano Taraglio, Enrico Mazza

**Affiliations:** ^1^General Surgery Unit, ASL Città di Torino, Turin, Italy; ^2^University of Turin, Sarcoma and Rare Visceral Cancer Unit, Turin, Italy; ^3^Endocrinology and Metabolism Unit, ASL Città di Torino, Turin, Italy; ^4^Pathology Unit, ASL Città di Torino, Turin, Italy

## Abstract

**Background:**

In 2014, the Italian Consensus for Classification and Reporting of Thyroid Cytology (ICCRTC) reviewed the previous cytological classification proposed in 2007 including the subdivision of TIR 3 category into low risk (TIR 3A) and high risk (TIR 3B). In Italian literature, different rates of malignancy have been correlated to these subcategories.

**Objectives:**

The aim of the study is to present our experience on this subclassification for the assessment of the malignancy risk of indeterminate thyroid nodules. We correlated the subdivision into TIR 3A and TIR 3B with the histological report by highlighting the rates of malignancy detected in the two subcategories. On the one hand, we aimed to check if the groups are associated with a real and significant difference risk of malignancy. On the other hand, we evaluated the use of this subdivision in the choice of the appropriate treatment.

**Study Design:**

This is a retrospective review of all the patients with an indeterminate nodule who underwent US-FNA and had surgery at ASL Città di Torino between January 2005 and May 2018.

**Results:**

150 patients have been analyzed for the research; 62 (41.3%) had a malignant histological report. Rates of malignancy between TIR 3A (20.8%) and TIR 3B (60.3%) were significantly different (*p* < 0.0001). The subclassification had high sensitivity (75.8%; CI 63.3–85.8%) and NPV (79.3%; CI 68–87.8%) and low specificity (64.8%; CI 53.9–74.7%) and PPV (60.3; CI 48.5–71.2%). The measurement of the accuracy (AUC = 0.7) classified the test as “moderately accurate.” *Conclusions*. Obtained data show a great rate of false negative (20.8%) and limited AUC (0.7). According to our logistic regression, we argue that the 2014 subclassification into TIR 3A and TIR 3B should be considered for the choice of patient treatment, but at the same time, we believe that the association with other screening tests is necessary to increase the accuracy in the future.

## 1. Introduction

Thyroid cancer is the most frequent endocrinological cancer, the 2.1% of all the diagnosis of cancer in the world [1]. In 2016 in Italy, patients with thyroid cancer were 160,307 [2]. The critical issue about the management of thyroid nodule is to distinguish preoperatively benign from malignant lesions (8–16%) [3] in order to make treatment most individualized.

Thyroid fine-needle aspiration (FNA) cytology is the most accurate and cost-effective screening and diagnostic tool used for distinguishing thyroid cancers [4]. Sensitivity of FNA cytology stands at 95% [4]. It improves preoperative diagnostic process and reduces unnecessary surgeries.

The limits of FNA are inadequate samples (5–10%), due to insufficient cellularity, and indeterminate reports (20–25%) [5, 6].

We must consider FNA cytology as a screening test. Final diagnosis is made by histological exam because what define malignancy is capsule or blood vessels invasion, not evaluable with cytological exam [4, 7].

To make communication between cytopathologists and physicians more efficient, in 2007, the Italian Societies of Endocrinology (AIT, SIE, and AME) and the Society of Pathology and Diagnostic Cytology (SIAPEC-IAP) created a “5-tiered TIR system” to classify FNA report of thyroid nodules [4].

In 2014, a team of experts of the abovementioned societies reviews the previous classification: nodules classified as TIR 3 have been divided into two subcategories with different risk of malignancy: TIR 3A (low risk) and TIR 3B (high risk) [8]. The 6-tiered system created in 2014 is represented in Table 1.

Analyzing previous Italian studies, we observed discrepancies in TIR 3A and TIR 3B malignancy rates: University La Sapienza of Roma reports a rate of 10.2% for TIR 3A nodules and 43.8% for TIR 3B (290 cases from 2008 to 2013) [9], University of Cagliari 21.1% for TIR 3A and 57.8% for TIR 3B (102 cases from 2014 to 2016) [10], and ASL Città di Torino 20.4% in TIR 3A and 53.8% in TIR 3B (96 cases from 2005 to 2015) [11].

Trimboli et al. [6] performed a meta-analysis of online articles until May 2018. The incidence of malignancy among the pooled series was 17% in TIR 3A category and 47% in TIR 3B.

Due to these different rates, we decided to analyze and present our experience.

## 2. Materials and Methods

### 2.1. Data Collection

**Figure 1:**
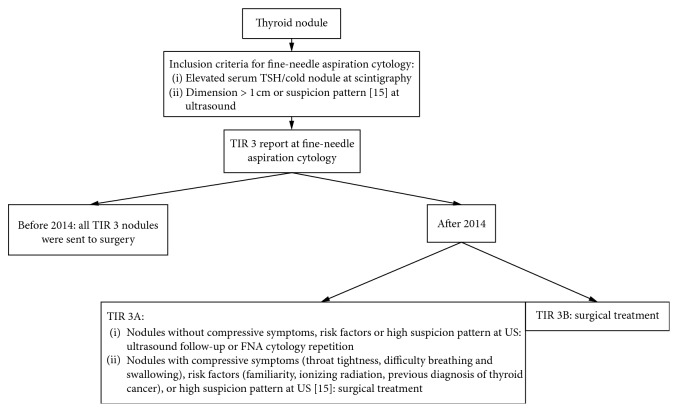
Flowchart: selection process of TIR 3 nodule.

This is a retrospective review of medical records of all the consecutive patients who underwent fine-needle aspiration cytology with ultrasound guide and thyroid surgery in a unique institution during a period of 13 years (from January 2005 to May 2018). A flow chart of the selection process of thyroid nodules is shown in Figure 1.

All the patients had a cytology report of TIR 3A and TIR 3B nodule, according to ICCRTC classification. The cytological and histological samples were analyzed by a unique pathology team.

Microscopic slides reported before 2014 were reanalyzed by the same team and subdivided into the two categories. Patients operated from 2005 to 2013 were 74/150 (49.3%), 34 were classified as TIR 3A lesions (47.2% of all TIR 3A in our study) and 40 as TIR 3B (51.3% of the TIR 3B).

Patients operated after 2014 were 76/150 (50.7%), 38 had a TIR 3A cytology report (52.8% of all TIR 3A), and 38 had a TIR 3B report (48.7% of all TIR 3B).

All FNAs were performed under ultrasound guide with a 21G needle. Slides adequacy was verified based on guideline of Papanicolaou Society [12] or Goellner Group [13]. All the samples were evaluated by our pathology team and reported according to 2014 ICCRTC classification.

TIR 3A category is characterized by specimen with increased cellularity and numerous microfollicular structures with low amount of colloid. This group also includes partially compromised specimens (due to preparation artifacts), with cytologic or architectural alterations that cannot be included in TIR 2 category [8].

TIR 3B group is characterized by a high cellularity in a monotonous and repetitive microfollicular/trabecular arrangement, with scant colloid. This subcategory also includes samples characterized by nuclear alterations suggestive of papillary carcinoma, too mild or focal to be included in TIR 4 category [8].

During samples analysis, pathologists were not aware of histological diagnosis in order to minimize bias.

### 2.2. Indication for Surgery

Before 2014, surgery was recommended to all the patients with a TIR 3 nodule. After 2014, just TIR 3B underwent surgery as first-line treatment [14]. In case of a TIR 3A nodule, we considered the presence of compressive symptoms (throat tightness, difficulty breathing, and swallowing), risk factors (familiarity, exposure of the neck to ionizing radiation, and previous diagnosis of thyroid cancer in contralateral lobe) and ultrasound properties (solid hypoechoic nodule or solid hypoechoic component of a partially cystic nodule with high suspicion characteristics such as irregular margins, microcalcifications, taller than wide shape, rim calcifications with small extrusive soft tissue component, and evidence of extra thyroid extension) [15].

In low-risk group, histologic data are available just for patients who underwent surgery treatment. For this reason, the rate of malignancy assigned to TIR 3A group represents only an estimation of the real risk.

In our study, 116 total thyroidectomies and 34 lobectomies were realized. Lobectomy was proposed to patients with ultrasound pattern not suggestive of malignancy, absence of compressive symptomatology, unilateral nodular disease, and regular function of contralateral lobe.

### 2.3. Statistical Analysis

Data were processed by Stata® software. To compare the malignancy rates in TIR 3A and 3B categories, the chi-squared test was used.

A ROC (receiver-operating characteristics) curve was created. We calculated the AUC (area under the curve) in order to measure the performance of the subclassification.

Subsequently, we realized a univariate and multivariate logistic regression analysis. By convention, it was fixed as level of significance *p* at 0.05.

## 3. Results

From January 2005 to May 2018, 150 patients underwent fine-needle aspiration cytology with a TIR 3A or 3B report and had surgery at ASL Città di Torino.

For each specimen, demographic (age and sex), and clinical data (nodule size and serum preoperative TSH) were obtained.

Patients were subdivided into TIR 3A and 3B category: 72 had a cytology report TIR 3A (48%) and 78 TIR 3B (52%). 62 patients (41.33%) had a final malignant histological report, considered as the Gold Standard of the diagnosis of thyroid carcinoma. In particular, we found 15 malignant diseases in TIR 3A category (20.83%) and 47 in TIR 3B (60.26%). Collected information of the analyzed population is shown in Table 2 while Table 3 shows the final histological diagnosis.

A 2 × 2 contingency table was created to compare TIR 3A and TIR 3B categories and calculate the main diagnostic indicators. The obtained values are shown in Table 4.

The prevalence of malignancy in TIR 3A category was 20.83% (15/72), whereas in TIR 3B group, it was 60.26% (47/78). The difference was statistically significant (*p* < 0.0001).

The two groups of patients (with and without cancer) were not different according to age (52.22 vs. 53.02 years, *t*=0.3487), sex (*p*=0.5032), nodule dimension (1.7 vs. 2.0 cm, *p*=0.8212), preoperative serum TSH (1.333 vs. 1.989 mU/L, *p*=0.0556), intranodal vascularity (*p*=0.681), and type of surgery (*p*=0.677).

Considering cytology categories (TIR 3A and TIR 3B), there is no difference between the two groups according to age (TIR 3A: 53.76 vs. TIR 3B: 51.71 years, *t*=0.9162), sex (*p*=0.2829), or nodule dimension (TIR 3A: 2 vs. TIR 3B: 1.6 cm, *p*=0.5825).

A logistic regression was performed to compare patients with and without malignant histological diagnosis and to evaluate the influence of clinical and demographic characteristics on the final diagnosis. In our study, we considered age, gender, preoperatory serum TSH, cytology, type of surgery, and intranodal vascularity, as shown in Table 5.

In univariate model, cytology is the unique variable that shows a significative influence on the histological report (*p* < 0.0001).

As previous univariate model, in multivariate logistic regression, cytology is the unique variable correlated to a significative influence on final histological diagnosis, as shown in Table 6.

## 4. Discussion

Indeterminate lesions represent the 20–25% of cytological reports of thyroid nodules [16].

It is a key issue to find an appropriate way to distinguish benign from malignant disease.

In our institution, the difference between TIR 3A (20.83%) and TIR 3B (60.26%) malignancy rates was statistically significant (*p* < 0.0001). Referring all patients with TIR 3A cytology to surgery would lead to an overtreatment in 4/5 of the patients, but it also means that 1/5 of the patients, who had a low-risk cytology and a malignant final diagnosis, could not be undertreated.

In our study, the malignancy rates are higher than the ones shown in the 2014 ICCRTC guidelines (TIR 3A < 10%, TIR 3B 15–30%) [8]. Our higher rates of malignancy in 3A group could be related to a different way of selection for TIR 3A lesions: after 2014, 3A nodules were sent to surgical treatment in case of compressive symptoms, risk factors, or ultrasound properties.

We believe that rates of malignancy of TIR 3B group are higher due to a meticulous selection of collection site (thanks to the use of ultrasound guide for FNA cytology) and anatomopathological analysis of the slides. Furthermore, Nardi et al. specify that expected rate of malignancy for the TIR 3 subcategories is mainly found on clinical experience and is only partially based on the evidence of the published data [8].

In our experience, the subclassification ICCRTC 2014 shows high sensitivity (75.8%) and negative predictive value (79.2%), but limited specificity (64.7%) and positive predictive value (60.3%). The high negative predictive value let detect a great number of benign lesions, but the low specificity and positive predictive value carried a significant number of false positive. These specimens are especially adenomatous lesions (considered benign disease) classified as high-risk cytology. Histology is the only way to differentiate correctly benign to malignant lesions [8, 17].

In our study, we do not take into account the presence of noninvasive follicular thyroid neoplasm with papillary-like nuclear features (NIFTP). It is a borderline entity with indolent clinical behavior [18]. As Trimboli et al. [6] underlined in their systematic review and meta-analysis, considering NIFTP, a benign histological outcome could decrease the rate of malignancy in both categories and the odds ratio of TIR 3B against TIR 3A, reflecting a predominance of this kind of lesion in TIR 3B category.

**Figure 2:**
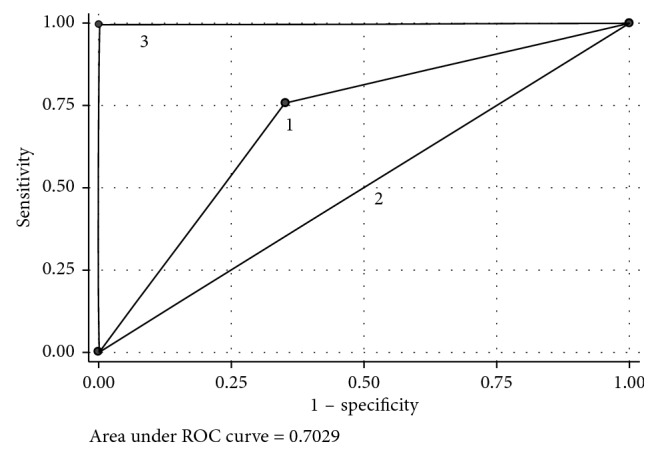
ROC curve of 2014 ICCRTC subclassification. (1) Accuracy of 2014 ICCRTC subclassification. (2) Reference line, corresponding to a useless test. (3) Excellent accuracy.

Then, we elaborated a ROC curve (receiver operating characteristic) and calculated the AUC (area under the curve) as a measure of diagnostic accuracy (Figure 2).

The accuracy of 2014 ICCRTC subclassification (represented by line number 1 in the figure) differs from the reference line (corresponding to a useless test and represented by line 2), but it does not reach the excellence (line 3). In our analysis, the subclassification shows an accuracy of 0.7 that classified the test as “moderately accurate.” Considering positive and negative likelihood ratios (LR (+) = 2.15; LR (−) = 0.374), their values demonstrate a small impact on the disease probability: the LR (+) shows an increase of malignancy probability of 15% in TIR 3B patients, while the LR (−) represents a reduction of the disease probability of 25% in TIR 3A patients. The test can be defined as “sometimes useful.”

To analyze confounding factors, we considered age, gender, preoperative TSH, nodule dimension, type of surgery, and intranodal vascularity in benign and malignant groups. No difference can be found between histology groups.

In the logistic regression, a significant result is obtained just for cytology (*p* < 0.0001). The result has been confirmed in the univariate and in the multivariate logistic regression model. None of the other analyzed variables reach a significative level (*p* > 0.05).

Although the logistic regression results and the different malignancy rates of the cytology categories, we believe that the association of further analysis is necessary in order to set the real malignancy risk. For example, the use of immunohistochemical markers can increase diagnostic accuracy with clinical and economic benefits. Galectin-3 and HBME-1 are the most commons markers used in thyroid FNA specimens. The association of both markers demonstrates higher accuracy and sensitivity than the use of a single one [19–21]. Although the results of these studies, immunohistochemical analysis are not used in clinical practice. 

Another useful tool in the management of thyroid indeterminate nodule could be the core needle biopsy (CNB). Pyo et al. [22] affirm that the CNB has a higher conclusive rate than the repetition of FNA cytology in nodule with atypia of undetermined significance/follicular lesion of undetermined significance (AUS/FLUS).

The limits of our study are its retrospective nature and the mixed analyzed population (made by patients treated before and after 2014). TIR 3A group includes patients surgically operated as a first-line treatment (from 2005 to 2013) and patients operated due to high-risk factors (from 2014 to 2018). The last group represents the 52.8% of TIR 3A patients (38/72). Because surgery indication of TIR 3A is not based on cytological report, low-risk indeterminate nodules treated after 2014 can be considered as a bias in the study. Nevertheless, a trial with a surgical approach to all TIR 3A patients is not ethical because of guidelines recommendation and surgically risks correlated to the treatment.

## 5. Conclusion

The aim of ICCRTC subclassification is to reduce unnecessary surgeries rate without underestimate malignant diseases.

The number of false negatives revealed by our study cannot be ignored (20.8%): 1/5 of TIR 3A patients could undergo an inappropriate treatment.

The sensitivity (75.8%) and specificity (64.8%) of 2014 subclassification founded in our study show limited values. Furthermore, diagnostic accuracy (represented by AUC) defines the test as mildly accurate. Likelihood ratios (LR (+): 2.15 *e* LR (−): 0.374) show a limited change in disease rates for TIR 3B reports.

Nevertheless, multivariate logistic regressions show as unique significative association the relation between cytology and final histological exam.

For these reasons, the 2014 subclassification can be considered as a helpful tool in the diagnosis of intermediate nodules, but we believe that the association with other screening tests will be necessary to increase the accuracy in the future.

Despite the small case series setup in our study, we hope our experience will be useful for a future improvement in the diagnostic process of thyroid nodules.

## Figures and Tables

**Table 1:** Diagnostic categories, expected risk of malignancy, and suggested management in 2014 ICCRTC classification [14].

	Diagnostic category	Expected rate of malignancy (%)	Recommendation
TIR1	Not diagnostic	Undefined	FNA repetition after at least one month
TIR1C	Not diagnostic, cystic	Low, based on clinical features	Focused on clinical features and/or FNA repetition
TIR2	Not malignant/benign	<3	Follow-up
TIR3A	Indeterminate nodule with low risk of malignancy	<10	Follow-up/FNA repetition
TIR3B	Indeterminate nodule with high risk of malignancy	15–30	Surgery
TIR4	Suspicious of malignancy	60–80	Surgery/intraoperatory histology eventually
TIR5	Malignant	95	Surgery; further diagnostic exams if necessary

**Table 2:** Demographic characteristics of the study population.

Cytology	Total	F	M	Mean age (years)	Malignant lesions	Mean dimension (cm)
TIR 3A	72	56	16	54 (25–79)	15 (20.83%)	2.65
TIR 3B	78	66	12	52 (22–84)	47 (60.26%)	2.23

**Table 3:** Final histological diagnosis associated with the relative cytological reports.

Histological diagnosis	TIR 3A	TIR 3B	Total
Adenoma	23	20	43
Papillary carcinoma	9	27	36
Follicular carcinoma	6	19	25
Poorly differentiated carcinoma	0	1	1
Medullary carcinoma	0	1	1
Goiter	27	9	36
Thyroiditis	7	1	8
*Total*	*72*	*78*	*150*

Malignant reports: follicular, papillary, poorly differentiated, oncocytic, and medullary carcinoma. Benign reports: adenoma, struma, and thyroiditis.

**Table 4:** Main diagnostic indicators of 2014 ICCRTC subclassification.

Sensitivity (IC 95%)	Specificity (CI 95%)	PPV (CI 95%)	NPV (CI 95%)	Likelihood ratio (+) (CI 95%)	Likelihood ratio (−) (CI 95%)	Odds ratio (CI 95%)	Area under ROC (CI 95%)
75.8 (63.3–85.8)	64.7 (53.9–74.7)	60.3 (48.5–71.2)	79.2 (68–87.8)	2.15 (1.57–2.95)	0.374 (0.234–0.596)	5.76 (2.8–11.9)	0.703 (0.629–0.776)

**Table 5:** Univariate logistic regression: analyzed variables and their respective *p* value.

Analyzed variable (univariate logistic regression)	*p* value
Age	0.726
Gender	0.504
Serum TSH	0.277
Cytology	0.0001
Type of surgery	0.677
Intranodal vascularity	0.681
Nodule dimension	0.388

**Table 6:** Multivariate logistic regression model.

Malignancy	Odds ratio	Standard error	*z*	*p*	CI 95%
Age	1.011098	0.0173889	0.64	0.521	0.977584–1.04576
Gender	1.788607	1.14382	0.91	0.363	0.5107035–6.264132
Dimension	1.183981	0.1845497	1.08	0.279	0.8723006–1.607028
Cytology	7.034768	3.568224	3.85	0.0001	2.603139–19.01088
Surgery	1.179955	0.6057121	0.32	0.747	0.4314333–3.227134
Intranodal vascularity	0.6030279	0.3193559	−0.96	0.340	0.2135758–1.70264
_cons	0.0693466	0.087607	−2.11	0.035	0.0058302–0.8248318

## Data Availability

The data used to support the findings of this study are available from the corresponding author upon request.
